# Validation of the Social Responsiveness Scale (SRS) to screen for atypical social behaviors in juvenile macaques

**DOI:** 10.1371/journal.pone.0235946

**Published:** 2021-05-20

**Authors:** Z. Kovacs Balint, J. Raper, V. Michopoulos, L. H. Howell, C. Gunter, J. Bachevalier, M. M. Sanchez

**Affiliations:** 1 Yerkes National Primate Research Center, Emory University, Atlanta, Georgia, United States of America; 2 Department of Pediatrics, Emory University, Atlanta, Georgia, United States of America; 3 Department of Psychiatry & Behavioral Sciences, Emory University School of Medicine, Atlanta, Georgia, United States of America; 4 Marcus Autism Center, Children’s Healthcare of Atlanta, Atlanta, Georgia, United States of America; 5 Department of Human Genetics, Emory University, Atlanta, Georgia, United States of America; 6 Department of Psychology, Emory University, Atlanta, Georgia, United States of America; Universita degli Studi di Parma, ITALY

## Abstract

Primates form strong social bonds and depend on social relationships and networks that provide shared resources and protection critical for survival. Social deficits such as those present in autism spectrum disorder (ASD) and other psychiatric disorders hinder the individual’s functioning in communities. Given that early diagnosis and intervention can improve outcomes and trajectories of ASD, there is a great need for tools to identify early markers for screening/diagnosis, and for translational animal models to uncover biological mechanisms and develop treatments. One of the most widely used screening tools for ASD in children is the Social Responsiveness Scale (SRS), a quantitative measure used to identify individuals with atypical social behaviors. The SRS has been adapted for use in adult rhesus monkeys (*Macaca mulatta*)–a species very close to humans in terms of social behavior, brain anatomy/connectivity and development–but has not yet been validated or adapted for a necessary downward extension to younger ages matching those for ASD diagnosis in children. The goal of the present study was to adapt and validate the adult macaque SRS (mSRS) in juvenile macaques with age equivalent to mid-childhood in humans. Expert primate coders modified the mSRS to adapt it to rate atypical social behaviors in juvenile macaques living in complex social groups at the Yerkes National Primate Research Center. Construct and face validity of this juvenile mSRS (jmSRS) was determined based on well-established and operationalized measures of social and non-social behaviors in this species using traditional behavioral observations. We found that the jmSRS identifies variability in social responsiveness of juvenile rhesus monkeys and shows strong construct/predictive validity, as well as sensitivity to detect atypical social behaviors in young male and female macaques across social status. Thus, the jmSRS provides a promising tool for translational research on macaque models of children social disorders.

## Introduction

Primates, both humans and nonhuman species, depend on social relationships that provide shared resources and protection critical for survival of the individual and the group. Social deficits present in neurodevelopmental disorders such as Autism Spectrum Disorder (ASD) and ADHD, and other psychiatric disorders (e.g. social anxiety, schizophrenia), can severely hinder the individual’s ability to function in a community. Social deficits impair daily functioning by altering social interactions, which can lead to social withdrawal and isolation [1]. The prevalence of ASD, in particular, is estimated at 1 in 59 children in the USA [2]. It is defined by impairments in social communication and interactions, repetitive/stereotypic patterns of behavior and restricted interests [3]. Children with ASD are also at high risk for anxiety disorders [4, 5] with an estimated co-frequency of ~40%, compared to ~6.5% in the general population [4]. Despite common reports of a 4:1 male:female ratio in diagnoses [3, 5], recent research suggests that ASD diagnosis in females using “gold-standard” instruments may be significantly underestimating prevalence, for a variety of sex- and gender-based factors including “compensatory camouflaging” learned through socialization [6, 7]. Early diagnosis and intervention is critical, given their positive effects in changing the trajectory of the disorder and improving outcomes [8, 9].

Identifying early biobehavioral markers of social deficits during development is critical for understanding ASD’s etiology and temporal unfolding, as well as for the development of early interventions and treatment. Currently, ASD can be diagnosed around 2 years of age (although the average age in the USA is 4 years old [2]), by observing deficits in social communication that develop as a child grows. The research and clinical standard to assess ASD and establish the diagnosis includes the Autism Diagnostic Interview–Revised (ADI-R), the Autism Diagnostic Observation Schedule (ADOS-2) and the Social Responsiveness Scale (SRS) [10–12]. The SRS, now in its 2^nd^ edition (SRS-2), is a widely used questionnaire that quantitatively measures the continuum of both typical and atypical social behaviors that covary with ASD symptom severity and is completed by the patient’s caregivers or a teacher [10, 13]. It is a regular ASD clinical and research tool that also identifies disruptive behavior disorders [14] and anxiety disorders co-morbid with ASD [5]. The full school-age SRS-2, for use between 4–18 years, consists of 65 questions, but a shorter SRS-2 has been developed and validated with 16 questions that measure ASD core symptom severity [15]. The SRS items measure a child’s ability (or deficits) to engage in reciprocal social interactions, and how deficits in communication and stereotypic/restricted interests and odd behaviors impair reciprocal social interactions in their naturalistic social environment [10]. Importantly, ASD deficits diagnosed with the SRS are different from and not captured by personality constructs/measures [16].

The complex nature of ASD has made it hard to study its etiology and biological roots in human populations. Therefore, true progress in understanding neurobiological mechanisms underlying ASD will require examination of those processes in nonhuman primate (NHP) models with sophisticated social behaviors, phylogenetic closeness to humans and brain anatomy, connectivity, function and development that closely resemble that of our species. Rhesus monkeys (*Macaca mulatta*), in particular, have been widely used to understand the typical development of human social behavior, including the evolutionary context for Bowlby’s “Attachment Theory” [17–19]. This species displays complex social behaviors, such as mother-offspring bonds, social play, reciprocal prosocial interactions, strong family alliances that preserve the status in the social hierarchy, as well as social awareness [20–23]. The complexity of rhesus monkeys’ social behavior, their physiological, anatomical and genetic closeness to humans, and parallels in brain and social development [24–32] make them an ideal model organism to study typical and atypical development of human social function.

To leverage rhesus monkeys as an NHP animal model of translational value for ASD-related social deficits, it is important to first develop and validate screening tools across species and developmental stages. With this goal in mind, researchers at the Yerkes National Primate Research Center (YNPRC) first adapted one of the most widely used screening tools for ASD, the SRS [6, 10], for use in adult rhesus macaques (*macaque SRS*: mSRS [33]). The mSRS tested the construct of social responsiveness in adult macaques through an adaptation of the *“Chimpanzee SRS”* which was, in turn, a cross-species adaptation of the human SRS to chimpanzees to measure social function [34]. The *“Chimpanzee SRS”* detected variation in social behavior and its factor structure resembled that of the human SRS. The adult mSRS adapted from chimpanzees by Feczko et al [33] also identified variability in social responsiveness, was sensitive to detect atypical social behaviors, and showed a factor structure similar to the human and the chimpanzee SRS. An additional advantage of the mSRS is that, like the human SRS–which can be filled out by a caregiver or teacher without previous knowledge about the test or about the social responsiveness construct–the mSRS does not require previous knowledge/training on the social responsiveness construct or test. In addition, the mSRS takes a relatively small amount of time to complete by coders following traditional behavioral observations of social behavior of the animals.

Unfortunately, the mSRS has not yet been validated or adapted for a necessary downward extension to younger ages matching those for ASD diagnosis in children. Since social deficits in ASD are rooted in early childhood, the development of a primate model to understand its etiology requires to first develop behavioral tools to identify typical and atypical social behaviors and underlying neurocircuitry in juvenile macaques. Therefore, the goal of the present study was to adapt and validate the adult mSRS to 16–18 months old juvenile macaques (males and females) with ages approximately equivalent to 5–6 yrs old school-age children (mid-childhood; e.g. [35]). Developing an instrument that can translate to existing children measurements of social deficits, quickly completed following behavioral focal observations, and complementing the quantitative behavior collected using more established ethograms in our laboratory [36, 37] is highly needed. Based on the results presented here, the juvenile mSRS (jmSRS) identifies variability in social responsiveness of juvenile rhesus monkeys and shows strong construct/predictive validity and sensitivity to detect atypical social behaviors in both male and female macaques across social status, providing a promising instrument for translational research on social disorders such as ASD.

## Materials and methods

### Subjects and housing

The subjects were 93 juvenile rhesus monkeys (*M*. *mulatta*) studied at approximately 1.5 yrs of age (average±SEM: 17.12 ±0.07mo, age range: 15.9–18.7mo; 75% data collected between 16.4–17.9mo of age), equivalent to human mid-childhood (e.g. [35]) and within the age range of the school-age human SRS-2 [15]. All animals lived with their mothers and families in complex social groups at the YNPRC Field Station breeding colony (Lawrenceville, GA). Of the 93 juveniles studied, 44 were females (social rank distribution: 18 high, 14 middle, 12 low) and 49 were males (social rank distribution: 15 high, 18 middle and 13 low) from six different social groups consisting of 55–130 adult females with their adolescent, juvenile, and infant offspring, and 2–4 adult males. At this young age, social rank of the juvenile is based on their mothers’ matrilineal social rank. Matrilineal social rank was assessed from aggression and submission behaviors exhibited during dyadic agonistic interactions during group observations with high rank defined as the top 33% most dominant, low rank as the lowest 33%, and middle rank as those in between. The groups were housed in outdoor compounds (approximately 100ft x 100ft) with access to indoor climate-controlled housing areas. Both the outdoor and indoor housing areas had physical structures to provide environmental enrichment for climbing/swinging/playing and shelter/shade. Animals were fed a standard commercial low‐fat, high‐fiber diet (Purina Mills International, LabDiets, St. Louis, MO) *ad libitum* in the morning and afternoon, supplemented twice each day with seasonal fruits and vegetables, and environmental enrichment items. Water was freely available. Subjects were excluded from the study if they were from an unstable social group, or had clinical conditions at time of assignment. Animals were monitored for health and well-being by animal care, colony management and/or veterinary staff 7 days per week, 24 hours per day (criteria for assessments of health and well-being: no signs of injury, illness or pain, including lethargy, depression -head down/crouching, motor retardation, social withdrawal-), unkept fur, etc. Upon completion of the studies, all animals were released back to the YNPRC breeding colony (i.e. no euthanasia was performed). All procedures were approved by the Emory University Institutional Animal Care and Use Committee (IACUC; protocol #201700546), and were performed in accordance with the Animal Welfare Act and the U.S. Department of Health and Human Services “Guide for the Care and Use of Laboratory Animals” [38]. The YNPRC is fully accredited by AAALAC International.

### Procedures

#### Behavioral data collection

Focal behavioral observations of the juveniles were collected by trained coders from observation towers situated over each social compound using a detailed ethogram adapted from a well-established rhesus monkey ethogram [39] and according to published methods [40–42]. The ethogram captured frequencies and durations of affiliative (proximity, grooming, eye gaze, touch), agonistic (aggression, submission), anxiety-like (yawn, body shakes) and play behaviors exhibited by each subject towards other group members (social, solitary), as well as time spent alone and atypical behaviors similar to those seen in ASD (e.g. stereotypies) and other species- and age-specific behaviors. The full list of coded behaviors and their operational definitions are presented in Table 1. Data were collected using netbook computers with an in-house data acquisition software (WinObs60) by four trained observers with an inter-rater reliability of Cohen’s k = 0.83. Subjects were identified by a distinctive dye-mark on their body (applied using non-toxic hair dye under ketamine anesthesia -10mg/kg body weight, i.m.-; animals were monitored continuously during anesthesia and every 5-15min post-anesthesia until recovery -sitting up, holding head up-). For a given subject, one coder collected all 4x30-min focal observations over several weeks (~4 wks) for a total of 2-hours of data per subject; at the end of the last (4^th^) focal observation, the same coder filled the jmSRS form for that animal (see next section). Observations were collected between 0700 and 1200 hr, when animals were most active. All animals in the social group were kept outdoors during the observation sessions.

**Table 1:** List of behaviors coded during the behavioral observations (ethogram).

Behavior	Description	Coded as
grooming	One animal combing through the hair of another, usually with hands, but can be with mouth.	duration
groom soliciting	Posture to solicit grooming from another animal, can groom solicit from more than one animal simultaneously.	frequency
proximity	Subject is within arm’s length to another animal. Not scored for animals passing by or when animals are in motion.	duration
eye-gaze	Making direct eye contact with another animal, if prolonged, 1 additional occurrence coded for every 3 seconds.	frequency
touch	Subject approaches and touches another animal, distinct from grooming; if prolonged, 1 additional occurrence coded for every 3 seconds.	frequency
social play	Playing with another animal, including wrestle, play chase and playing with tale.	frequency
contact	Majority of body is touching another animal.	duration
solitary play	Does not involve a partner, vigorous play by oneself. Manipulate/tactile an object (e.g. rock, poop, cage), climb or swing for at least 3 seconds.	duration
sitting alone	Sitting out of proximity (arm’s length) to other animals.	frequency
aggression	Hostile interaction between 2 animals, with or without physical contact between the animals (e.g.: slap or grab and attack, bite, physical threat, open mouth, barking, lunge).	frequency
scream	High pitch, high intensity screech or loud chirp.	frequency
submissive behavior	Sum of lip-smacking (opening and closing lips) and withdrawing (avoiding or pulling away from another animal) behaviors.	frequency
grimace	Open mouth wide to show teeth with a closed jaw to another animal, pulling back of lips to display teeth.	frequency
display	Shaking or bouncing vigorously to convey dominance status.	frequency
following	Persistent trailing of another animal; both moving simultaneously, within arm’s reach.	duration
breaking proximity	Leaving behind another animal (beyond arm’s reach), end of being in proximity (within arm’s length) with another animal.	frequency
anxiety	Sum of scratching, yawning, body-shaking and self-directed behaviors (e.g. self-exploring, self-grooming).	frequency
sex-related behavior	Sum of hip touch (subject places two hands on hip of the other animal) and mount (subject’s feet are clasped on the outside of the ankles of the other animal) behaviors.	frequency
stereotypy	Any odd/repetitive patterns of behavior.	duration

Two hours of focal behavioral data was collected from each animal (4x30min sessions on 4 different days) in their social compound, based on the ethogram above.

#### Juvenile macaque social responsiveness scale (jmSRS)

A 14-item global rating instrument of typical and atypical social behaviors was used for our juvenile macaques. This jmSRS instrument was adapted from the final 17 items retained as “reliable and pertinent questions” in the adult rhesus macaque SRS (mSRS; [33]), which initially tested the 36 chimpanzee SRS items [34]. For this study, only 14 out of the 17 published adult mSRS questions [33] were selected, based on high intra-item reliability and after eliminating the questions not applicable for the juvenile age or considered too subjective or anthropomorphic for coding–*“Wanders aimlessly from one activity to another”*; *“Touches others in an unusual way”*; *“Shows indiscriminate grooming”*- (Table 2). Similar to our 14 item jmSRS, a short form of the human school-age SRS-2 has been developed and validated with 16 questions [15]. In addition, the items were rated on a 5-point Likert scale (1 = not true 0%, 2 = sometimes true 25%, 3 = often true 50%, 4 = almost always true 75%, and 5 = always true 100%), instead of the 4-point scale used by Feczko and colleagues [33] to provide higher resolution in the ratings. For each juvenile macaque, the 14-item jmSRS form was completed once, after the last (fourth) 30min focal observation; that is, after 2 hrs of behavioral data collection/animal, spread over 4 weeks. The same coder completed all 4x30min focal observations and the jmSRS on a given animal, limiting the examination of jmSRS inter-rater reliability in this dataset. Given the high day-to-day individual variability in behavior, our rationale was to allow each coder to collect as much behavioral information for each juvenile across different days (2 hours) before filling in the jmSRS. It took ~5-10min to fill in the 14-item jmSRS form and the four coders completed all focal observations and jmSRS independently.

**Table 2:** List of items included in the jmSRS and criteria for scoring based on the 1–5 likert rating scales.

For the following statements, select the score that best represents the subject based on your current observations. Leave any comments if you feel you cannot accurately rate the statement or have other concerns.
**1** = not true (0%), **2** = sometimes true (25%), **3** = often true (50%), **4** = almost always true (75%), **5** = always true (100%)
1. *Seems self-confident when interacting with others*. *R*
2. Would rather be alone than with others.
3. Behaves in ways that seem strange or bizarre for others of comparable age/rank/gender categories.
4. Is not well coordinated in physical activities.
5. *Responds appropriately to other monkeys’ vocalizations and facial expressions*. *R*
6. Avoids eye contact or has unusual eye contact.
7. *Plays appropriately with peers*. *R*
8. Avoids social interactions with others.
9. Is socially awkward.
10. Has a restricted or unusually narrow range of interests.
11. Has repetitive, odd behaviors such as hand flapping, rocking/swaying, tumbling or spinning.
12. Is too tense in social situations, e.g., walks siffly, stiffens or freezes when others approach.
13. Stares or gazes off into space.
14. *Manifests species and status-typical reaction to loss of a valued resource*. *R*

The jmSRS was adapted from the adult mSRS [33]. *R*: reverse-coded items (after data collection, the scoring of these items was reversed, so that higher scores meant greater social impairment for each item).

However, we were able to calculate jmSRS inter-rater and test-retest reliability from a different group of juveniles (n = 28) at the YNPRC. High inter-rater reliability was found using Cronbach’s alpha between two experienced coders (Cronbach’s alpha = 0.798). In addition, significant test-retest reliability was detected for two expert coders using intraclass correlation coefficients (ICC = 0.650, p = 3.45x10^—8^).

### Data reduction and analyses

JmSRS data reduction and analyses of distribution, demographics, internal reliability and validity followed previously published methods by our group to develop and validate an instrument for global maternal quality rating in rhesus monkeys [41].

#### Descriptive statistics of the jmSRS

Prior to analysis of the jmSRS data distribution, demographics, internal reliability and validity, four items were reverse-coded to match the interpretation of higher scores meaning greater social impairment by the rest of the items: (1) “Seems self-confident when interacting with others”, (5) “Responds appropriately to other monkeys’ vocalizations and facial expressions”, (7) “Plays appropriately with peers”, (14) “Manifests species and status-typical reaction to loss of a valued resource”. Total scores for each subject were calculated as the sum of all items, with possible scores ranging between 14 and 70 (based on 14 items with a 1–5 Likert scale score/each). The individual distribution of the total jmSRS scores were plotted and the Kolmogorov-Smirnov test was used to analyze the shape and distribution of the total scores.

#### Analysis of jmSRS internal reliability

Exploratory factor analysis (EFA) was performed using IBM SPSS Statistics 26 to determine the overall factor structure of the jmSRS 14 items and for data reduction. “Principal axis factoring” was used as the extraction method, and the “latent root criterion” (factors with eigenvalues >1) was used to establish the number of factors. Promax rotation method was then used to achieve simpler and theoretically more meaningful factor solutions. Factor scores were then calculated for each subject individually, using the factor score method; factor loadings >0.4 were considered high.

After the factor structure of the jmSRS was determined using EFA, the internal reliability of each factor was tested using Cronbach’s α analysis. The internal consistency of a factor was considered high if α ≥0.7, moderate if 0.5 ≤ α < 0.69, or unacceptable if α < 0.5.

#### Analysis of jmSRS validity

In order to assess the convergent construct and discriminant validity of the jmSRS, we examined the associations between each jmSRS EFA factor score and the individual social and non-social behaviors collected during the two hours of focal observations with the ethogram. For that, the frequency or the duration of each behavior was first summed per subject across the two hours of behavioral observations, and then frequency rate or duration proportion per hour was calculated for each behavior. When the Kolmogorov-Smirnov normality test failed (p<0.05), behavioral data were Z-scored. Then, to test the validity of the jmSRS, Spearman correlation coefficients were calculated to examine the associations between the jmSRS factor scores and frequency rates or proportion of time spent on behaviors collected during the focal behavioral observations. Correlation coefficients were calculated between behaviors in Table 1 (except sitting alone and stereotypic behaviors, which were excluded due to low occurrence [<15%]) and the jmSRS factor scores. For significant associations, Spearman correlations were re-run excluding outliers (defined as 3SD above or below the mean, and representing <4.5% of our population), to ensure that significant findings were not driven by these outliers. For the correlation analyses, significance level was set at p < 0.05.

#### Analysis of sex and social rank

Multivariate ANOVA was used to examine the effects of SEX (male, female) and social RANK (high, medium, low) on the jmSRS factor scores. A parallel multivariate ANOVA was performed on behavioral data collected with our ethogram to confirm expected SEX and social RANK differences in this species. Before the ANOVAs, the Kolmogorov-Smirnov test was performed to examine the normal distribution of both jmSRS factor scores and behavioral frequency and duration rates; data were log-transformed when normality failed (p<0.05). Post-hoc pairwise comparisons were performed with Bonferroni adjustments, when main or interaction effects were detected. Significance level was set at p<0.05.

## Results

### Descriptive statistics of the jmSRS

**Fig 1:**
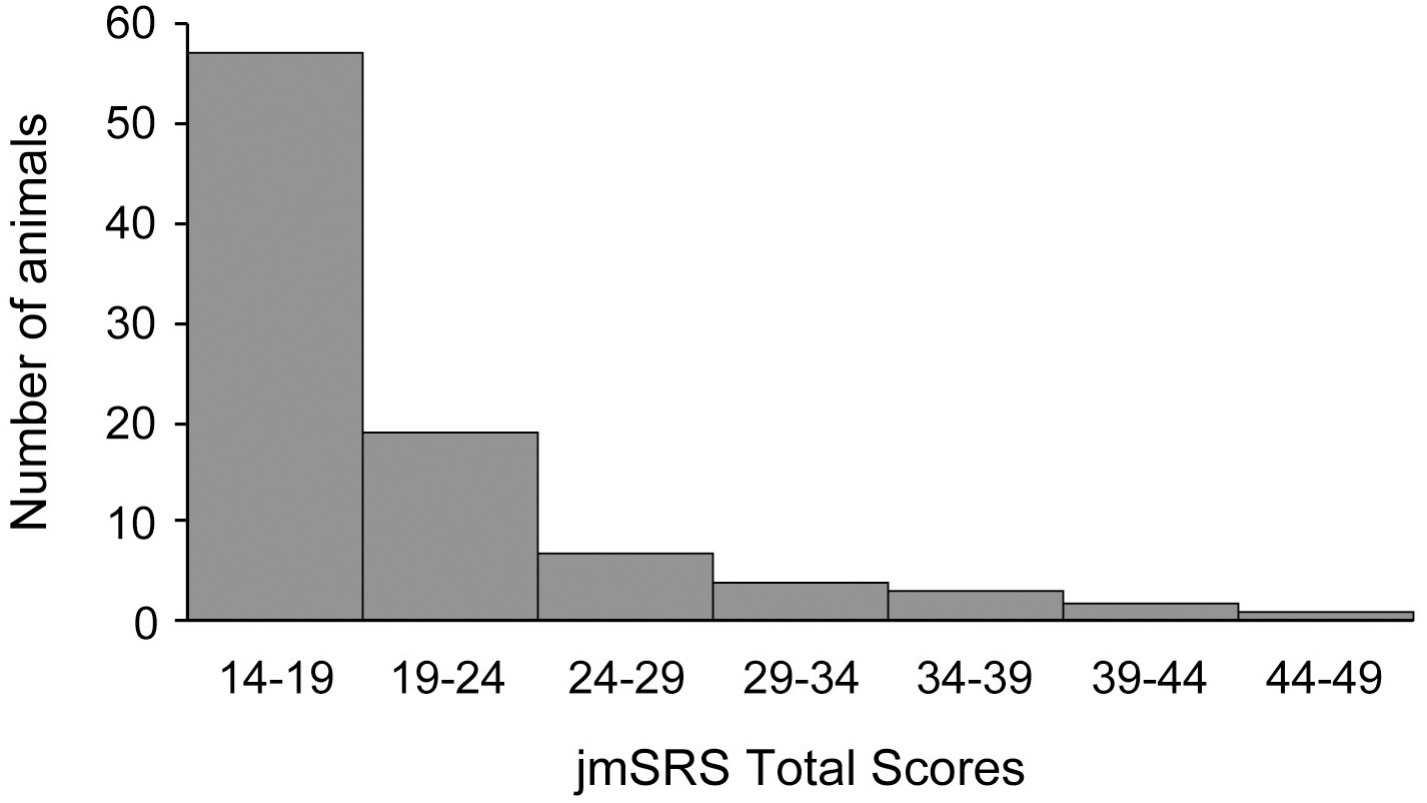
Histogram showing data frequency distribution of the jmSRS total scores in our juvenile macaque population. The jmSRS total score was calculated for each subject as the sum of scores for each jmSRS item. Each column represents the number of animals with the jmSRS total score indicated on the X-axis.

First, we calculated the sum of scores (total scores) for each animal. Scores ranged between 14 and 48, with a mean of 19.98 and a median of 18. The Kolmogorov-Smirnov analysis revealed that the total scores were not normally distributed (KS = 0.192, p = 4.56x10^-9^), and that the distribution was positively skewed (skewness = 1.889, kurtosis = 3.805), indicating that a few animals (6.5%) had very high total scores (between 34–48; i.e. they were low in typical social behaviors and/or high in atypical behaviors). Fig 1 shows the distribution of the jmSRS total scores.

### Analysis of jmSRS internal reliability

The jmSRS EFA revealed a solution in which the first four factors explained approximately 68% of the variance in the data, accounting for 39.41%, 10.24%, 9.48% and 8.55%, respectively. In this 4-factor EFA solution (Table 3), jmSRS Factor #1 explained the majority of the variance -similarly to the human and chimpanzee SRS and the adult mSRS [33, 34, 43]. The jmSRS Factors #1 and #2 together contained items that matched the first criterion domain of human ASD (deficits in social communication and social interactions [3, 10, 43, 44]) in the human SRS Factor 1, as well as items related to social avoidance/awkardness and social anxiety in the mSRS and chimpanzee SRS Factor 1 (e.g. *“Avoids social interactions with others”*, “*Would rather be alone than with others”*, *“Socially awkward”*; *“Is too tense in social situations”*). Thus, our first two factors encapsulated the items/domains previously combined in just one first factor, which seem to measure the same construct across species and macaque ages [33, 34, 43]. Items loading on Factor #3 were related to impaired motor coordination and staring off into space; while items in Factor #4 matched the second diagnostic criteria of ASD (repetitive/stereotypic behaviors [3, 10, 43, 44], and bizarre behaviors. Each item in the jmSRS had a high factor loading (>0.4) on at least one factor (Table 3), and only item 3 (*“Behaves in ways that seem strange or bizarre for others of comparable age/rank/gender categories”*) showed significant loadings on more than one factor (cross-loading on Factors #2 and #4), supporting the choice of this 4-factor EFA solution as the best underlying relationship structure between items; this also suggests more segregation of items into 4 distinct behavioral domains in the jmSRS than in the “unitary factor solution” of the human, chimpanzee and adult mSRS.

**Table 3:** Exploratory factor analysis (EFA) of the 14 jmSRS items: 4-factor solution.

	jmSRS Factor			
	#1	#2	#3	#4
**Item 1**. *Seems self-confident when interacting with others*. *R*	0.474	−0.124	0.334	0.007
**Item 2**. Would rather be alone than with others.	0.787	0.029	0.091	−0.024
**Item 7**. *Plays appropriately with peers*. *R*	0.56	0.101	0.028	0.174
**Item 8**. Avoids social interactions with others.	0.945	−0.019	0.039	−0.226
**Item 10**. Has a restricted or unusually narrow range of interests.	0.568	0.072	0.051	0.231
**Item 14**. *Manifests species and status-typical reaction to loss of a valued resource*. *R*	0.578	−0.005	−0.397	0.237
**Item 3**. Behaves in ways that seem strange or bizarre for others of comparable age/rank/gender categories.	−0.068	0.418	0.23	0.54*
**Item 5**. *Responds appropriately to other monkeys’ vocalizations and facial expressions*. *R*	0.072	0.51	−0.138	0.112
**Item 6**. Avoids eye contact or has unusual eye contact.	−0.123	0.99	−0.145	−0.057
**Item 9**. Is socially awkward.	0.326	0.544	−0.029	−0.062
**Item 12**. Is too tense in social situations, e.g., walks siffly, stiffens or freezes when others approach.	0.275	0.566	0.137	−0.196
**Item 4**. Is not well coordinated in physical activities.	0.066	−0.28	0.623	0.165
**Item 13**. Stares or gazes off into space.	−0.002	0.155	0.688	−0.007
**Item 11**. Has repetitive, odd behaviors such as hand flapping, rocking/swaying, tumbling or spinning.	0.011	−0.115	0.089	0.643

EFA revealed a 4-factor solution that explained approx. 68% of the variance in the data, and had high item factor loadings (criteria: >0.4). Gray numbers indicate items with low factor loadings (<0.4).

*: Significant cross-loading on Factors #2 and #4, *R*: reverse-coded items.

**Fig 2:**
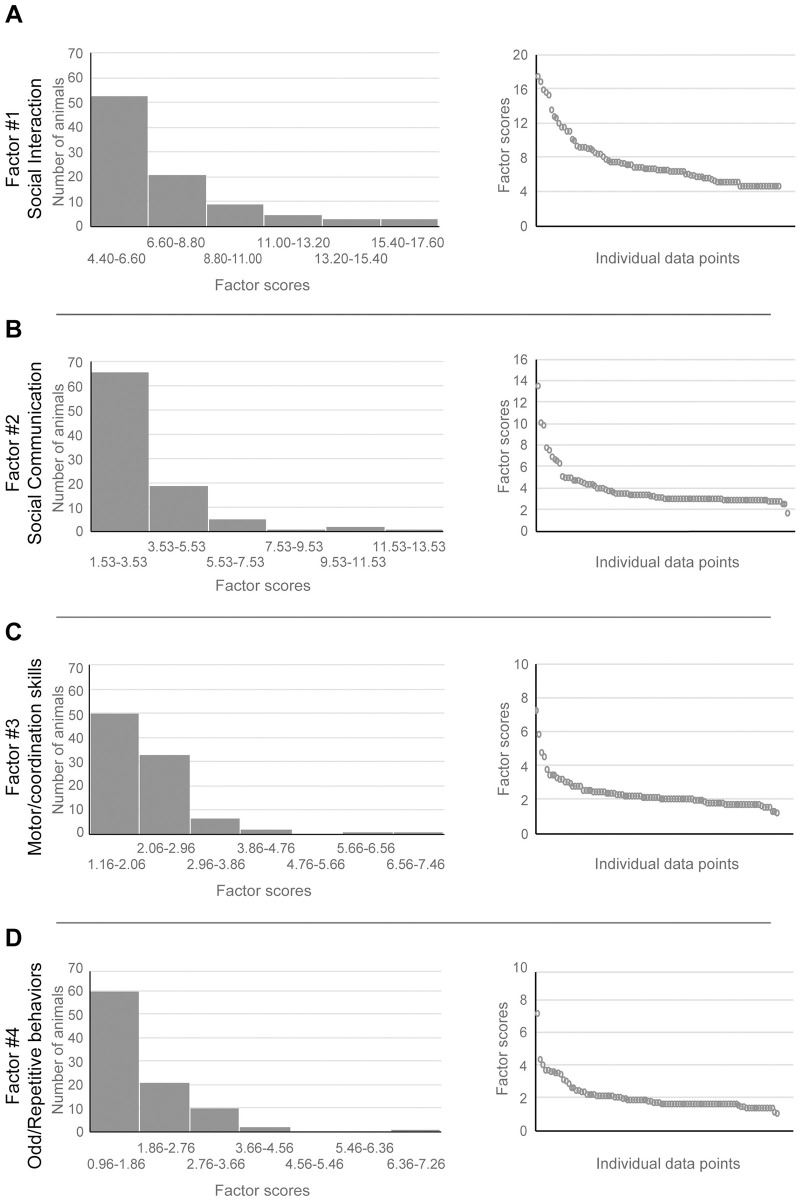
JmSRS EFA factors scores distributions. **Left**: Histograms showing frequency distribution of jmSRS Factors #1, #2, #3 and #4’s scores (calculated by multiplying the item score by the factor loading per subject) in our juvenile macaque population. **Right**: Individual Factor scores, each circle represents a subject (n = 93). **A)** jmSRS Factor #1 (Social interaction); **B)** jmSRS Factor #2 (Social communication); **C)** jmSRS Factor #3 (Impaired motor coordination, staring off into space); **D)** jmSRS Factor #4 (Repetitive/Odd behaviors).

Kolmogorov-Smirnov tests showed that the jmSRS EFA factors scores were not normally distributed (jmSRS #1: KS = 0.196, p = 1.87x10^-9^; jmSRS #2: KS = 0.261, p = 2.68x10^-17^; jmSRS #3: KS = 0.208, p = 1.16x10^-10^; jmSRS #4: KS = 0.231, p = 2.49x10^-13^), and that the distribution of each factor score was positively skewed (jmSRS #1: skewness = 1.621, kurtosis = 2.328; jmSRS #2: skewness = 3.082, kurtosis = 11.624; jmSRS #3: skewness = 2.96, kurtosis = 11.975; jmSRS #4: skewness = 2.962, kurtosis = 12.730). Fig 2 shows the distribution of the jmSRS factor scores and the individual data.

Six out of 14 items showed high factor loadings on Factor #1, and 5 on Factor #2, which together represented the first criterion domain of ASD (social communication and interest), based on the DSM-V [3]. Items loading on Factor #3 reflected impaired motor coordination and eye-gaze patterns (*“Is not well coordinated in physical activities”*, *“Stares or gazes off into space”*). Items loading on Factor #4 matched the second criterion domain of ASD as defined in the DSM-V (restrictive, repetitive behavior and interest [3]). The factor score distributions along all four factors were positively skewed. That is, although the jmSRS factors had enough sensitivity to rate animals in a behavioral continuum, most subjects had low scores -indicating typical social behaviors/responsiveness-, and only a few animals were detected with high scores -indicating low typical and high atypical social behaviors- (see Fig 2A–2D).

To test the internal (inter-item) consistency or reliability within each jmSRS factor, Cronbach α values were calculated. The analysis revealed good reliability for Factors #1 and #2, based on high Cronbach α (0.825 and 0.803, respectively), low reliability for Factor #3 (0.278) and moderate reliability for Factor #4 (0.521). Since only 2 items loaded on Factors #3 and #4, this could explain the lower Cronbach α values.

Overall, the results of the EFA with a good 4-factor solution that explains approximately 68% of the variance, items with high factor loadings, and the high/acceptable Cronbach’s α results suggest appropriate internal reliability of the four factors created from the 14-item jmSRS questionnaire.

### Analysis of jmSRS validity

**Fig 3:**
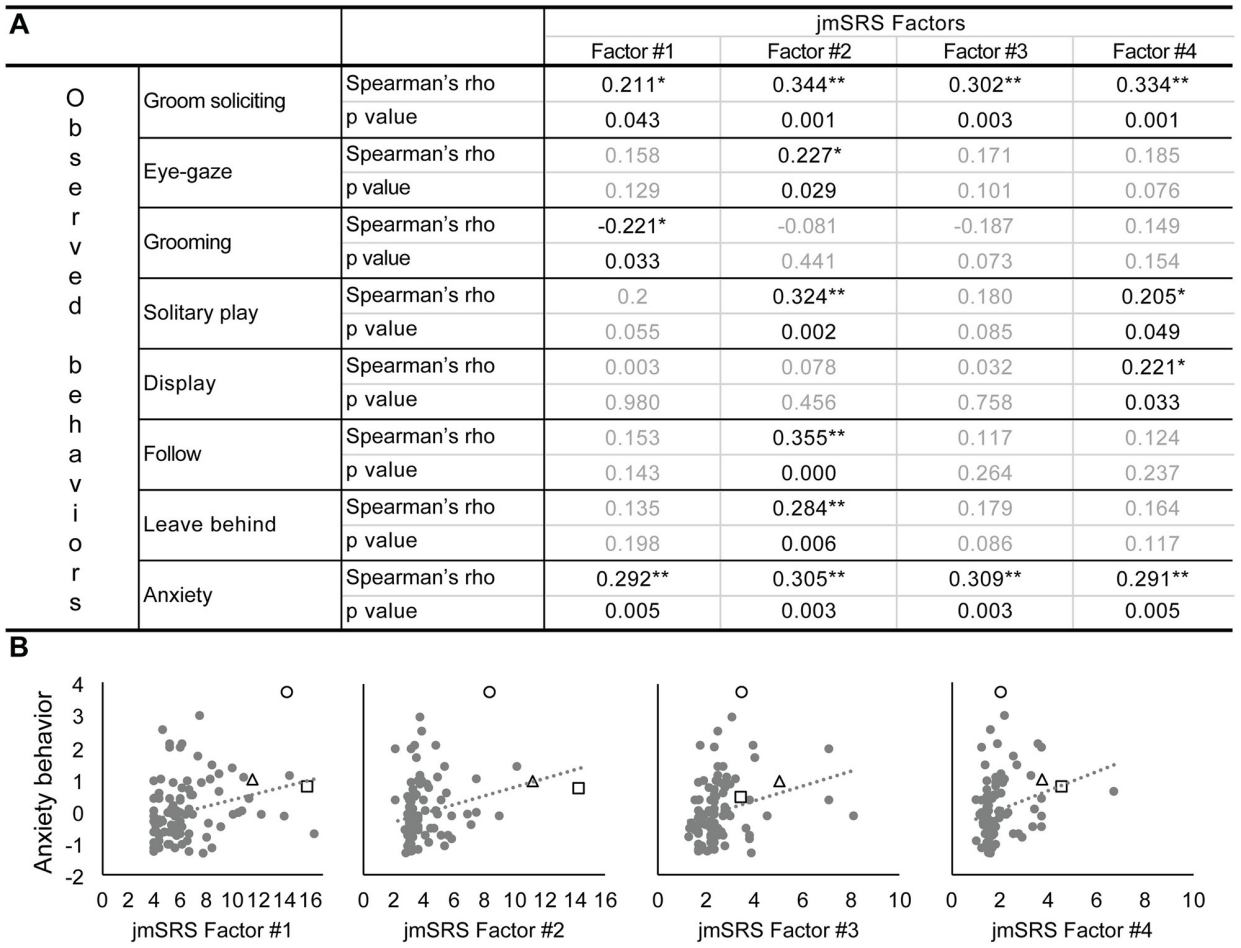
Correlations between the jmSRS factors (#1, #2, #3, #4) and behavior frequencies and durations collected with the ethogram. **A)** Correlation matrix, showing significant Spearman rho correlation coefficients between Ethogram behaviors and the jmSRS Factors. *p<0.05, ** p<0.01. **B)** Examples of regression plots representing individual Anxiety and jmSRS Factors score data. Y-axis represents *Anxiety Z-scored frequency rates (counts/hour; Anxiety frequency is calculated as a composite score -sum- of frequencies of scratching*, *yawning*, *body-shaking and self-directed behaviors such as self-grooming)*; X-axis represents the jmSRS factor scores (Factors #1–4) for each individual. The triangle, square and circle symbols represent the same 3 subjects in each graph, marked as outliers in the jmSRS and/or the *anxiety* behavioral scores.

Results of the correlation analysis between the jmSRS factors scores and the observed (ethogram-based) behaviors are shown in Fig 3. JmSRS Factor #1 -which consists of items measuring impairments in social interactions/play- showed significant positive correlations with *anxiety* (rho = 0.292, p = 0.005) and *groom soliciting* (rho = 0.211, p = 0.043), whereas it was negatively correlated with *grooming* behavior (rho = -0.221, p = 0.033). JmSRS #2 -which represents items measuring atypical/awkward social communication/responses- showed significant positive correlations with *anxiety* (rho = 0.305, p = 0.003), *groom soliciting* (rho = 0.344, p = 0.001), *eye-gaze* (rho = -0.227, p = 0.029), *solitary play* (rho = 0.324, p = 0.002), *leave behind* (rho = 0.284, p = 0.006) and *following* behaviors (rho = 0.355, p = 4.86x10^-4^). JmSRS #3 -with items related to impaired motor coordination and staring off into space (which has been reported in children with ASD)- was also positively correlated with *anxiety* (rho = 0.309, p = 0.003) and *groom soliciting* (rho = 0.302, p = 0.003). JmSRS #4 -which represents items related to repetitive/stereotypic, odd behaviors- was also positively correlated with *anxiety* (rho = 0.291, p = 0.005), *groom soliciting* (rho = 0.334, p = 0.001), *solitary play* (rho = 0.205, p = 0.049) and *display* behaviors (rho = 0.221, p = 0.033). Most significant correlations still held after removing outliers from the analyses (defined as 3 SD above and below the group mean, representing <4.5% of the population), except for 4 associations that seemed driven by outliers: JmSRS #1 vs. groom soliciting and grooming; jmSRS #3 vs. groom soliciting; and jmSRS #4 vs. display (see S1 Table for details).

These correlations suggest that impairments in social responsiveness in juveniles (i.e. high scores in the jmSRS Factors #1, #2, #3 and #4) are associated with higher rates of anxiety, solitary play, and also breaking proximity with (*“leave behind”*) other animals. The significant correlations with social behaviors collected with the ethogram demonstrate the convergent/construct validity of the jmSRS instrument, and will be interpreted in more detail below, in the Discussion.

### Analysis of sex and social rank

Based on the results of the Kolmogorov-Smirnov test described above, showing that the jmSRS EFA factor scores were not normally distributed, multivariate ANOVA was run in log-transformed data. Results of the ANOVA showed no main SEX (jmSRS #1: F(1,84) = 2.987, p = 0.09; jmSRS #2: F(1,84) = 0.074, p = 0.79; jmSRS #3: F(1,84) = 0.154, p = 0.70; jmSRS #4: F(1,84) = 0.019, p = 0.89) or RANK effects (jmSRS #1: F(2,84) = 0.662, p = 0.52; jmSRS #2: F(2,84) = 0.522, p = 0.60; jmSRS #3: F(2,84) = 0.673, p = 0.51; jmSRS #4: F(2,84) = 0.607, p = 0.55) or SEX x RANK interaction effects (jmSRS #1: F(2,84) = 0.191, p = 0.83; jmSRS #2: F(2,84) = 0.325, p = 0.72; jmSRS #3: F(2,84) = 0.448, p = 0.64; jmSRS #4: F(2,84) = 0.221, p = 0.80). No significant SEX, RANK or SEXxRANK interaction effects were found for the total jmSRS scores, either (data not shown).

In contrast, a parallel multivariate ANOVA performed on behavioral data collected with our ethogram confirmed the expected SEX and Social RANK differences in this species. Because the Kolmogorov-Smirnov test revealed non-normal distribution of the Z-scored behavioral data, and log-transformation could not be performed due to some behavioral scores being negative or “0”, we used the Greenhouse-Geisser-corrected results. The ANOVA revealed significant SEX differences for the following behaviors: social play (F(1,84) = 9.172, p = 0.003, males>females), sex-related behavior (F(1,84) = 32.264, p = 1.88x10^-7^, males>females), submissive behavior (F(1,84) = 6.53, p = 0.012, males<females) and fear grimace (F(1,84) = 8.777, p = 0.004, males<females), and a trend for breaking proximity (F(1,84) = 3.37, p = 0.07, males<females). We also found significant RANK effects for the following behaviors: aggression (F(2,84) = 5.376, p = 0.006, high>middle>low rank animals) and submissive behavior (F(2,84) = 15.548, p = 2x10^-6^, low>middle> high rank animals). Significant SEX x RANK interaction was observed for proximity behavior (F(2,84) = 5.136, p = 0.008), with more time spent in proximity for high ranking males than females, whereas the opposite was observed in middle ranking animals.

## Discussion

The goal of this study was to adapt and validate the adult mSRS to assess social and non-social behaviors in 16–18 months old juvenile macaques of equivalent ages to human mid-childhood. We termed this downward extension of the instrument the “jmSRS”, composed of 14 items that measure global dimensions of typical and atypical social behaviors, as well as stereotypic and odd behaviors of relevance to ASD in juveniles. The EFA analysis identified 4 jmSRS factors with high levels of internal consistency/reliability and items with high factor loadings along the constructs identified in the human SRS as relevant to ASD: jmSRS #1) impairments in social interactions/play; #2) atypical/awkward social communication/responses; #3) impaired motor coordination and staring off into space; #4) stereotypic/repetitive and odd behaviors. The jmSRS factors #1 and #2 matched the first criterion domain of human ASD (deficits in social communication/interactions [3]); #4 matched the second diagnostic criteria of ASD (repetitive/stereotypic behaviors [3]) and odd behaviors. The jmSRS identified variability in social responsiveness in juvenile macaques and showed sensitivity to detect individuals with low typical and high atypical social behaviors in both males and females, and across social ranks. Finally, the significant associations between impairments in social responsiveness in the 4 jmSRS factors (i.e. high scores) and higher rates of anxiety, solitary play, and proximity breaking with other animals collected with the well-established behavioral ethogram further support the convergent/construct validity of the jmSRS instrument; similar associations have been reported in ASD in children with the human SRS. Our findings indicate that the jmSRS can be easily filled by trained primate researchers, complementing standard focal observations with social responsiveness data critical for future screening in macaque models of ASD.

The human SRS is a widely used rating scale to quantitatively measure the continuum of symptom severity in ASD in children [10]. This test, now in its 2^nd^ edition (SRS-2), is commonly used with other diagnostic tools to assess of symptom severity in ASD in children, but rarely in adults [13]. The complex and neurodevelopmental nature of ASD has made it hard to study its biological roots in children. Thus, the study of NHP models including the rhesus monkey, a species that closely resembles humans in terms of socially complex behavior, brain anatomy/connectivity and development, is crucial to understand neurobiological mechanisms underlying ASD. Cross-species adaptations of the human SRS have been published in chimpanzees [34] and rhesus macaques (mSRS; [33]); however, both studies focused on adapting the SRS to adult populations. Given that the early diagnosis and assessment of ASD symptom severity is crucial for intervention in children and infants, this study adapted and validated the adult mSRS for juvenile male and female macaques of all social status/ranks at approximately 1.5 yrs, which is roughly equivalent to mid-childhood in humans, with the goal of using this screening instrument to identify atypical social, motor and other behaviors of relevance for ASD in children among socially-housed juvenile macaques.

The jmSRS instrument is a 14-item rating scale adapted from the final 17 items published for the adult mSRS [33], which initially tested the 36 items in the chimpanzee SRS [34]. The 14 jmSRS items were selected out of the 17 adult mSRS questions [33] based on high intra-item reliability, applicability to juveniles and objectivity for coding. Similar to our 14-item jmSRS, a short form of the human school-age SRS-2 has been developed and validated with 16 questions [15]. Trained observers scored the social behavior of 93 juvenile rhesus macaques using the jmSRS, in parallel to traditional behavioral observation data collected using a well-established ethogram [39–41]. The distribution of the jmSRS total scores (sum of scores of all items per subject) was positively skewed, similarly to the adult mSRS [33] and the human SRS [44–46]. This result indicates that very few animals (6.5%) showed very high total jmSRS scores -between 34 and 48- (i.e. they were high in atypical behaviors and low in typical social behaviors), while most juveniles showed high typical and low atypical social behaviors, which is consistent with the human SRS observations [45]. Thus, similarly to the human, chimpanzee and adult mSRS the jmSRS is a screening tool with enough sensitivity to rate individuals in a continuum of social, motor and other behaviors of relevance to ASD, from “typical” to “atypical”, while identifying individuals with extreme high scores (high in atypical and low in typical social behaviors).

The exploratory factor analysis (EFA) revealed a strong 4-factor solution that explained approximately 68% of the variance in the data, with high items’ factor loadings and high to acceptable internal consistency/reliability. Factors #1 and #2 represented items that matched the first major criterion domain of ASD -deficits in social communication/interactions-, based on the DSM-5 [3]: #1) impairments in social interactions/play, and #2) atypical/awkward social communication/responses, accounting for the vast majority of the variance (39.41% and 10.24%, respectively), similarly to the first factor in the human SRS (34.97%), chimpanzee SRS (52.27%) and adult mSRS (30.64%). Thus, our first two jmSRS factors encapsulated items and behavioral domains previously combined in just one first factor that measures a similar construct across species and macaque ages [33, 34, 43]: social interactions/communications. Factor #3 (9.48% of the variance) represented items related to impaired motor coordination–consistent with recent reports of impaired motor coordination, postural control and balance in children with ASD [47–49] and staring off into space–also consistent with reports in children with ASD [50]. Factor #4 (8.55% of the variance) included items represented in the second diagnostic criteria of ASD (restricted, repetitive/stereotypic patterns of behavior, interests or activities [3] and odd behaviors.

The jmSRS 4-factor structure recapitulates what earlier studies revealed for the human, chimpanzee and mSRS, except for a key difference: There is more segregation of items into 4 distinct behavioral domains in the jmSRS than in the “unitary factor solution” of the human, chimpanzee and adult mSRS [33, 34, 43]. While the jmSRS Factors #1 and #2 are related to the first main ASD criterion domain and Factor #4 is related to the second main ASD criterion domain, in the human, chimpanzee and adult mSRS both criterion domains are included in just one first factor [33, 34, 43]. Regardless, items loading strongly on our two first jsmSRS factors or in Factor #1 in human, chimpanzee and mSRS are all related to social communication and interactions (e.g. *“avoids eye-contact”*, *“avoids social interaction”*, *“socially awkward”*), which confirms that this first factor(s) reliably measures a similar social construct across primate species [10, 33, 34]. This observation highlights the translational value of the instrument for ASD in humans.

The distribution of the jmSRS factor scores were also positively skewed, with the vast majority of the subjects showing low scores, indicating species-typical and age-appropriate social behavior. Yet, a few subjects scored high on the jmSRS (6.5%), supporting the sensitivity of this jmSRS instrument to identify extreme animals based on their atypical social, motor and stereotypic/odd behavior. The lack of sex or social rank effects on the jmSRS factor scores, despite confirmation of expected sex and social rank differences in behavioral data collected with our ethogram [40, 42] was surprising, particularly given the association between higher scores and lower rank in the adult mSRS [33], the chimpanzee SRS [34], and the male:female higher ratio of children with ASD deficits [2, 10]. It is possible that atypical social behaviors detected with the jmSRS are, indeed, independent of sex and rank at this juvenile age because animals are still immature. Future longitudinal studies with larger samples sizes need to further examine these relationships.

The significant associations between impairments in social responsiveness, motor coordination and stereotypic/repetitive/odd behavior in the jmSRS factors (i.e. high scores) and higher rates of anxiety and low sociability collected with the well-established behavioral ethogram support the convergent/construct validity of the jmSRS instrument; similar associations have been reported in ASD in children with the human SRS. Subjects scoring higher on the jmSRS Factor #1 (i.e. deficits in social interactions/play) were more anxious and groomed other animals less, although they solicited grooming more. Higher scores in Factor #2 (atypical/awkward social communication/responses) were also associated with higher rates of anxiety, solitary play, breaking proximity with other animals (leave behind), but the animals also seemed to seek more attention from others (they solicited more grooming, and followed others and eye-gazed more). Under normal circumstances, grooming behavior in this species emerges around five months of age and increases with age [23, 51]. Infants practice grooming behavior on their mother first, which later gets generalized to other family members (aunts, cousins), and then to peers and other animals in the subject’s environment [23, 51]. Playing behavior in macaques exhibits a similar developmental increase, beginning around 7–12 weeks of age followed by a rapid increase in the frequency until it peaks near the end of the first year, which coincides with the age at which our subjects were observed [23, 51]. Thus, decreased amounts of grooming and increased amounts of non-social (solitary) play suggest lower sociability, which confirms the construct validity and sensitivity of the first two jmSRS factors to identify deficits in social communication and interactions.

Subjects with higher scores on Factor #3 (impaired motor coordination, stares off into space) were also more anxious and solicited more grooming. Impaired motor coordination, postural control and balance has been reported in children with ASD [47–49], as well as staring off into space [52, 53]. Finally, subjects with higher scores on Factor #4 (exhibiting repetitive/stereotypic and odd behaviors) were also more anxious, played alone for longer periods, solicited more grooming and exhibited more display behavior. It is important to note that increased anxiety and proximity/attention-seeking are not necessarily unexpected in animals with low typical social behaviors. In fact, some ASD individuals initiate and participate in social interactions, but their overtures or responses are not appropriate to the situation and can generate anxiety [4, 5]. As in humans, failure to reach social connection (i.e. inappropriate following, groom-soliciting, proximity) could generate anxiety on its own.

The associations between high scores in the 4 jmSRS factors and elevated anxiety are very interesting, given that children with ASD have consistently shown to be at high risk for anxiety disorders [4, 5]. In fact, anxiety disorders are the second most common comorbid conditions in people with ASD -behind ADHD- (see meta-analysis: [4]), with current consensus on co-frequency estimated around 40% in children/adolescents with ASD, compared to a consensus of 6.5% in the general population [4]. Both “deficits in social interaction and communication” [54, 55] and “restrictive and repetitive behaviors” [56–58]–which are the two major criteria for ASD diagnoses [3]–have been associated with increased levels of anxiety. A recent study that analyzed the association between social responsiveness using the human SRS and anxiety in 150 pre-adolescents and adolescents with ASD, also showed strong correlations between the SRS total score and anxiety, and between the “Social communication” and “Autistic mannerisms” subscales of the SRS and anxiety [59], which is consistent with the associations we report here between high scores in all 4 jmSRS factors and higher juvenile anxiety. Altogether, the associations reported support the jmSRS construct validity and sensitivity to identify atypical social behaviors of relevance for ASD. Thus, our findings suggest that the jmSRS is a promising translational tool for studies in developing macaque models of children ASD.

The goal of this study was to adapt and test the validity of the jmSRS, a downward extension of the adult mSRS [33] to juvenile-age macaques, as a potential sensitive instrument that provides measures of atypical social, motor and stereotypic behaviors, similarly to the human SRS used for ASD diagnosis in children [10, 43]. The ultimate goal is to develop and apply this translational tool to identify social deficits of relevance for ASD in socially-housed macaque NHP models. Developing translational NHP models of ASD-related social alterations is critical, and such models must show similar patterns and complexity of brain and social development to humans.

The study also has some limitations that need to be considered. First, we were not able to address the intra-rater or test-retest reliability in the jmSRS scores in this dataset. We tried to overcome that limitation recruiting expert macaque behavior coders that filled in the jmSRS questionnaire after completion of all 4 x 30 min focal behavioral observations based on our ethogram (so they were very familiar with juvenile macaque -and each subjects’- social behavior), and their inter-rater reliability for those focal observations was high (Cohen’s k >.80). In addition, we provided intra-rater and test-retest reliability in a separate dataset. However, future studies need to address both the jmSRS inter- and intra-rater reliability across all coders in the same sample. Additional studies are also needed to adapt the jmSRS instrument to measure typical and atypical behaviors in younger–infant- monkeys and longitudinally, from infancy through adulthood, using within-subject designs.

In summary, this study adapted a downward extension of the adult mSRS, and tested its external validity and sensitivity to identify atypical social, motor and stereotypic behaviors of relevance to ASD in socially-housed juvenile rhesus macaques. Our findings with the jmSRS instrument show high levels of internal reliability, sensitivity to detect individuals with atypical behaviors and convergent construct validity, supported by the associations between the jmSRS factors and the behavioral observation data. Based on the similarities found with the human SRS, the jmSRS is a promising translational tool for studies in developing macaque models of ASD in children.

## Supporting information

S1 Dataset De-identified dataset. (XLSX)

S1 Table Correlations between the jmSRS factor scores and the observed behaviors, after removing outliers from the analyses. Correlation matrix, showing significant Spearman rho correlation coefficients between behaviors and the jmSRS Factors (#1, #2, #3, #4) after removing outliers from the analyses (subjects 3SD above or below the mean, representing <4.5% of our population). *p<0.05, ** p<0.01.
(PDF)
