# Supplementary material for: Validation of the Social Responsiveness Scale (SRS) to screen for atypical social behaviors in juvenile macaques
Source: PLoS One. 2021 May 20;16(5):e0235946. doi: 10.1371/journal.pone.0235946 (PMC8136728; doi:10.1371/journal.pone.0235946)
Supplement: S1 Table — Correlation matrix, showing significant Spearman rho correlation coefficients between behaviors and the jmSRS Factors (#1, #2, #3, #4) after removing outliers from the analyses (subjects 3SD above or below the mean, representing <4.5% of our population). *p<0.05, ** p<0.01. (PDF) [file pone.0235946.s002.pdf]

			jmSRS Factors			
			FACTOR #1	FACTOR #2	FACTOR #3	FACTOR #4
Observed Behaviors	Groom soliciting	Spearman's rho	0.200	0.358**	0.195	0.258*
		p value	0.060	0.001	0.067	0.015
	Eye-gaze	Spearman's rho	0.129	0.22*	0.174	0.093
		p value	0.230	0.039	0.102	0.385
	Grooming	Spearman's rho	-0.176	0.013	-0.160	0.136
		p value	0.094	0.901	0.132	0.200
	Solitary play	Spearman's rho	0.172	0.304**	0.154	0.238*
		p value	0.103	0.004	0.147	0.023
	Display	Spearman's rho	-0.040	-0.038	0.036	0.190
		p value	0.710	0.723	0.738	0.074
	Follow	Spearman's rho	0.124	0.34**	0.094	0.097
		p value	0.245	0.001	0.378	0.361
	Leave behind	Spearman's rho	0.162	0.322**	0.175	0.153
		p value	0.122	0.002	0.096	0.146
	Anxiety	Spearman's rho	0.257*	0.231*	0.278**	0.269**
		p value	0.014	0.029	0.008	0.01
